# Linking international technical specifications for acoustic characterization of marine energy converter sounds with environmental compliance criteria

**DOI:** 10.1038/s41598-026-52491-x

**Published:** 2026-08-12

**Authors:** Joseph Haxel, Xiaoqin Zang, James McVey, Carola Chicco, Emma Cotter, Marta Picciulin, Garrett Staines

**Affiliations:** 1 Coastal Sciences Division, Pacific Northwest National Laboratory, Sequim, 98382 Washington, United States; 2 Earth Systems Science Division, Pacific Northwest National Laboratory, Richland, 99352 Washington, United States; 3 Department of Environmental, Land and Infrastructure Engineering, Politecnico di Torino, Torino, 10129 Italy; 4 Marine Offshore Renewable Energy Lab, Politecnico di Torino, Torino, 10129 Italy; 5 Institute of Marine Sciences, National Research Council, CNR-ISMAR, Venice, 30122 Italy; 6 Department of Mechanical Engineering, University of Washington, Seattle, 98195 Washington United States

**Keywords:** Marine energy, Wave energy converter, Underwater sound, Environmental effects, Ecology, Ecology, Environmental sciences, Ocean sciences

## Abstract

As new ocean energy technologies emerge and are deployed for testing and operations, sound emissions are a potential concern for environmental effects to marine life. Consistent acoustic measurement and data analysis methods can help promote comparisons of technologies and transferability between project sites. In 2022, acoustic emissions from a prototype scale wave energy converter (WEC) were characterized for a range of environmental conditions and power generation states in the coastal waters off southern California using a set of international technical specifications. Results from the international technical specification analyses were applied to United States regulatory threshold criteria for acoustic impacts to marine mammals and examined in the context of European underwater noise monitoring guidelines. Weighted 24 hour cumulative sound exposure levels $SEL_{24h}$ calculated from the highest WEC power generation state were often more than 20 dB re 1μPa^2^·s below threshold criteria for temporary threshold shifts in five relevant marine mammal hearing groups. Following European Union recommendations for analyses and reporting, WEC sound characterization in third octave bands centered at 63 Hz and 125 Hz show clear spatial decay of WEC-generated noise, with more pronounced attenuation at 63 Hz, and a less marked but still detectable gradient at 125 Hz, collectively suggesting a relatively confined acoustic footprint under the observed conditions. The value of the international technical specification approach is highlighted by the isolation of WEC sounds from the surrounding soundscape. This allows for a robust characterization of acoustic emissions through a range of device power generation and sea states. Furthermore, in threshold-based regulatory contexts like the U.S., this facilitates direct evaluation of source contributions, while in broader monitoring frameworks used in the E.U. it provides a reproducible foundation for assessing the contribution of emerging ocean energy technologies to the underwater acoustic environment.

## Introduction

Harvesting power from the ocean is an important initiative for meeting global energy demands, ensuring energy security, providing energy equity, and reducing emissions. However, the spectrum of demonstrated capabilities for converting marine energy to electricity falls significantly short of the potential natural energy resources available in open ocean and tidal areas. For instance, an estimated 57% of the United States (U.S.) energy needs from 2019 could be met with marine energy sources, yet typically only 10% of marine energy resources are assumed technically available using the current power conversion technologies^1^. To close this technology gap, rapid, iterative, deployment and testing of the full range of readiness levels of marine energy converters (MECs) in a variety of high energy coastal and open water settings is critical. Yet, the introduction of novel renewable energy technologies and infrastructure has raised environmental concerns for some coastal area stakeholders. Uncertainties around the potential environmental effects of MECs often result in a conservative regulatory approach. This can lead to significant delays and higher project costs that act as barriers to device deployments and negatively impact marine energy project success and subsequent industry growth.

Concerns about the potential environmental effects of underwater noise produced by MECs have been consistently raised by community stakeholders and regulators in the consenting process^2^. Since many marine mammals^3,4^, fishes^5–7^, and invertebrates^8^ use sound for a variety of important life functions, the acoustic conditions of ocean habitats are critical for healthy marine ecosystems^4,9^. Thus far, there is no evidence that sounds from single or small numbers of operational MECs could cause mortality or acute auditory injury to marine animals. Rather, concerns are centered around arrays of devices and the potential for sound emissions to mask important biological or behavioral acoustic cues^10^ or disrupt vital behaviors^11,12^, impacting the long-term health and success of marine animals in these important habitats. Furthermore, device sound emissions may also contribute to coastal soundscapes that are already largely influenced by anthropogenic sources^13–15^. The lack of information resulting from a dearth of consistent, standardized measurements of underwater noise from MECs is a challenge for regulators responsible for managing these coastal areas^16^. There is particular uncertainty surrounding the acoustic emissions from wave energy converters (WECs), a class of MECs that transform kinetic and potential energy from ocean waves to mechanical or electrical energy, because there are a broad range of WEC device types and operating principles that affect the sound generated by a device. As a result, regulators often consider noise effects from WECs as having high uncertainty and high risk in the permitting process^17^.

In 2019, the International Electrotechnical Commission (IEC) published technical specification 62600-40, “Marine Energy – Wave, tidal and other water current converters – Part 40: Acoustic characterization of marine energy converters”, hereto referred to as the −40 TS^18^. The −40 TS details uniform methodologies to characterize sound produced by marine energy converters including specifications for sensors and sample rates, recording durations, hydrophone deployment methods and spatial configurations or sampling areas, and relevant co-temporal meteorological and oceanographic data. Two levels of acoustic characterization are described: Level A, which has higher spatial and temporal detail; and Level B, which has reduced spatial and temporal detail, but requires less effort and cost. The measurements performed in the use case provided in this paper are most closely aligned with the specifications for a Level A characterization of a WEC, which calls for measurements from three fixed hydrophones over the course of 6 months or sea states corresponding to 50% of the WEC’s annual energy production (AEP). A Level A characterization for WECs provides the added value of documenting sound emissions through a variety of environmental and power production states which is lacking in Level B characterization.

While the −40 TS provides detailed guidance as an engineering specification for acoustic characterization of WECs, including how data should be collected and analyzed, it does not discuss how these measurements should be interpreted in the context of effects on animals, a critical component for environmental regulatory decision making and project compliance monitoring. Yet, acoustic regulatory criteria for marine animals vary internationally, making standardized conversion of the −40 TS engineering characterization of sound emissions to animal effects thresholds for regulatory compliance challenging. In this paper, we use acoustic recordings from a WEC to connect the −40 TS characterization with regulatory criteria from the U.S. National Marine Fisheries Service (NMFS) regulatory guidance for understanding the impacts of measured anthropogenic sound on marine mammals^19^; furthermore, we discuss the results in the context of the European Union (EU) Marine Strategy Framework Directive (MSFD, 2008/56/EC)^20^. This directive requires Member States to achieve Good Environmental Status (GES) and has driven coordinated European research to develop underwater noise monitoring programs and to define thresholds relevant to GES for underwater noise^21,22^.

Prior studies that have characterized the sound produced by WECs have used a wide variety of data collection methods and have reported different metrics, making comparison between studies challenging^16^. For instance, several studies have employed free drifting hydrophone systems, with the earliest documented study conducted around a one-seventh scale heave and surge point absorber WEC in Puget Sound, Washington, USA^23^. Acoustic characterization data was collected at ranges of 10 m – 1,500 m and acoustic frequencies from 20 Hz-20 kHz. Similarly, an attenuator WEC was characterized at the European Marine Energy Center using hydrophones cabled to a free-drifting vessel with the propulsion system turned off to avoid contamination^24^. Power spectral density calculations of received levels showed similar trends (although lower in amplitude) to bottom-mounted, fixed hydrophones located closer to the WEC. At the U.S. Navy and University of Hawaii Wave Energy Test Site (WETS), a point absorber WEC was also characterized with drifting hydrophones, and sound emissions from the power generator were observed between 50 Hz – 300 Hz^25^. The highest amplitude acoustic signals, detected in frequencies up to 5 kHz, were associated with mooring system components. Most recently, in 2024, advanced drifting hydrophone systems^26^ were used to characterize the acoustic emissions from the same WEC that was tested in Puget Sound in 2011, but at a larger scale and rated power capacity for open water testing in the open ocean at WETS. Using an array of drifting hydrophones, signals could be localized and attributed to specific components of the WEC at a limited range (up to 150 m) and remained below 120 dB re 1 $\mu {Pa}$ during the operational conditions and frequency band where WEC sound was detected (60–900 Hz)^27^. Notably, the larger scale WEC was quieter due to engineering improvements informed by the previous study in 2011.

In addition to free drifting hydrophones, sound emission characterizations of operational WECs have also used fixed hydrophones on the seafloor or suspended in the water column. In an early study, a hydraulic point absorber was characterized using a fixed hydrophone moored 25 m from the WEC off the Danish North Sea coast^28^. The majority of WEC noise, described with median sound pressure levels, occurred in the frequency range 125–250 Hz with received sound pressure levels (*SPL*) 1–2 dB above ambient conditions. A study conducted off the coast of Portugal collected fixed hydrophone acoustic measurements around a semi-operational, seabed-anchored oscillating surge WEC^29^. Broadband WEC noise was observed from 50 Hz-20 kHz, with the peak in received levels occurring at a frequency of 125 Hz. Again, mooring system components noise was a significant sound source and a confounding signal for characterizing WEC generated sounds in the ambient field. The same WEC was further characterized at a test site in the United Kingdom^30^ with a hydrophone mounted on the seafloor at a distance of 200 m. A source level of 155 dB re $\mu {Pa}$ at 1 m was estimated from those recordings with tonal peaks occurring at 30 Hz and 60 Hz attributed to the WECs power generator. Moreover, in the Mediterranean Sea, a study reported that full-scale WEC operations generated continuous low-frequency noise, predominantly below 4 kHz, with dominant spectral components under 100 Hz. Third-octave band sound pressure levels at 63 Hz reached approximately 126 dB re 1 $\mu {Pa}$ at a distance of 40 m from the WEC^31^. Lastly, a bottom mounted acoustic vector sensor and hydrophone platform collected data at ranges of 100 m and 200 m from a WEC deployed off the southern California coast USA during low energy environmental conditions^32^. WEC-attributed sound emissions were observed in frequencies 200 Hz - 2.7 kHz with an estimated sound exposure level (SEL) of 139 dB re 1 $\mu \textrm{Pa}^2\,\textrm{s}$.

Measurements of operational WEC sound emissions have shown the acoustic energy is primarily observed below 1 kHz and is consistently lower amplitude than other anthropogenic disturbances of regulatory concern (e.g. seismic exploration, pile driving, vessel noise). Nevertheless, the diversity in hardware, methodologies, analysis, and reporting of WEC characterizations has made cross-comparisons and device specific evaluations of acoustic emissions difficult. Adoption of the −40 TS by researchers in future acoustic characterizations of WECs and other marine energy converters has the potential to alleviate inconsistencies in measurement and analysis, providing a standardized approach. Furthermore, connecting −40 TS characterization to environmental regulatory threshold criteria and reporting metrics that conservation managers and decision makers are familiar with is a critical next step for permitting and licensing of marine energy deployments.

In this study we present acoustic data collected from three seafloor hydrophones deployed near a WEC tested off the coast of La Jolla, California. Data collection and analysis follow the Level A −40 TS to characterize WEC sound emissions through different power production states and environmental conditions. We then interpret these results in the context of two sets of regulatory criteria: the U.S. National Marine Fisheries Service (NMFS) Technical Guidance for Assessing the Effects of Anthropogenic Sound on Marine Mammal Hearing^19^ and the E.U. Marine Strategy Framework Directive (MSFD) descriptors 11.1 and 11.2. While the NMFS guidance focuses on auditory impacts to marine mammals, the MSFD descriptors provide a framework to evaluate the impact of underwater noise on indicator species and ecosystems^33^. The results not only provide valuable environmental effects information for a growing marine energy industry, but also demonstrate the utility and value of the −40 TS.

## Results

### WEC acoustic characterization from the −40 TS

**Fig. 1:**
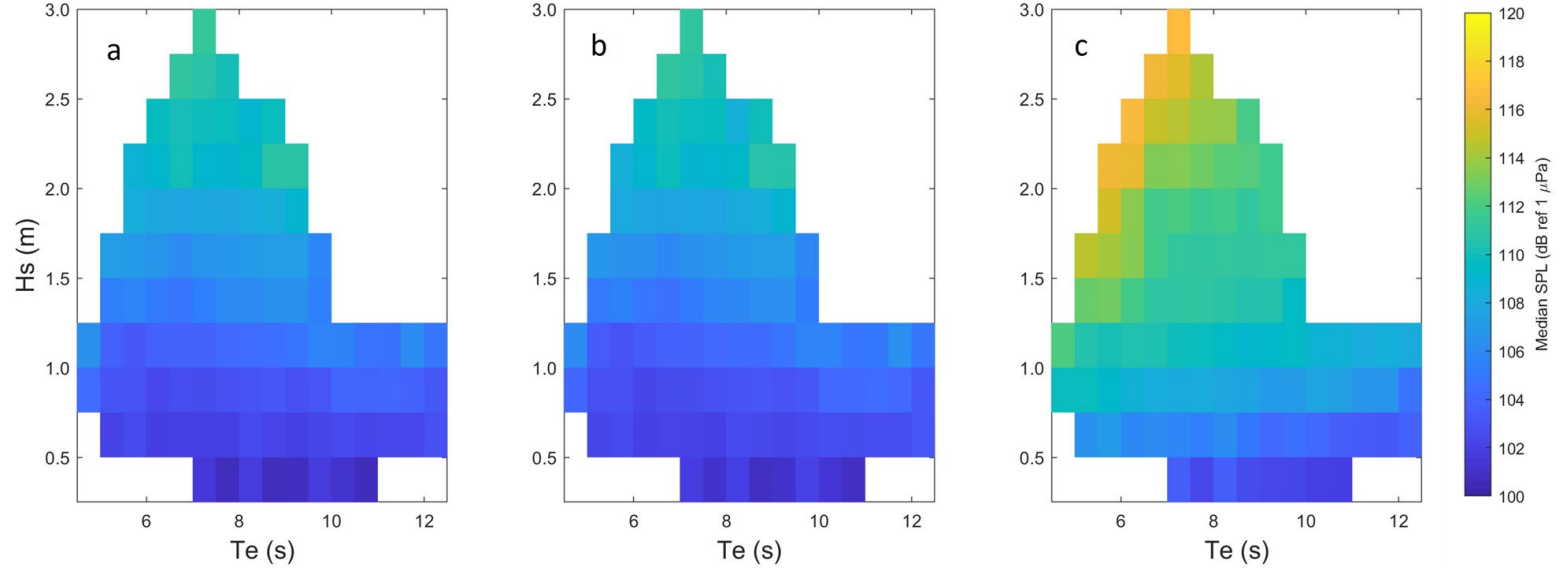
Median sound pressure levels (10 Hz - 96 kHz) as a function of significant wave height $H_s$ and wave energy period $T_e$ at: (**a**) Station 1 (110 m range), (**b**) Station 2 (170 m range), and (**c**) Station 3 (10 m range). Wave heights and periods are binned into 0.25 m and 0.5 s windows, respectively.

**Fig. 2:**
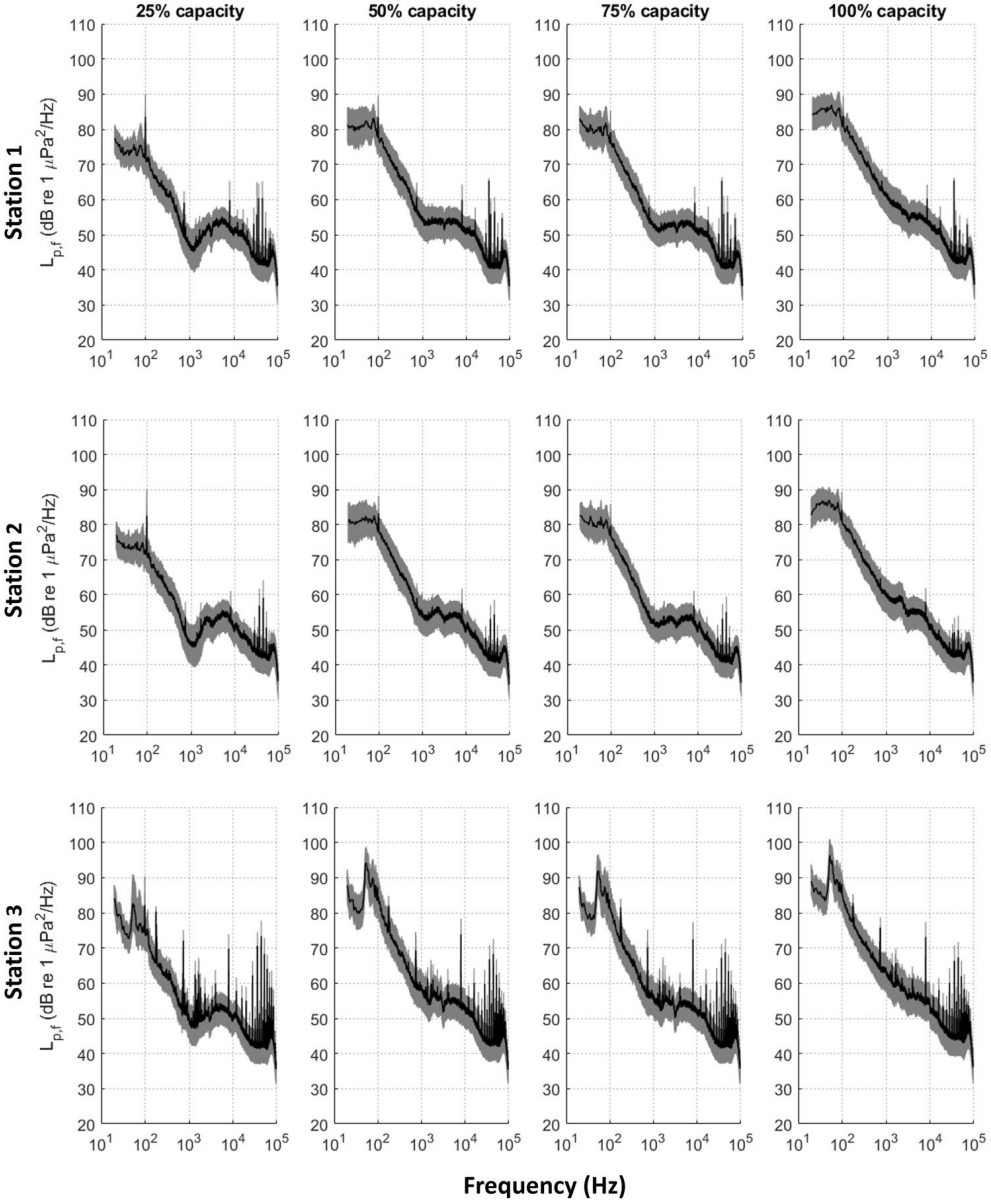
Median values (black curves) and interquartile range (gray shadow) of the mean-square sound pressure spectral density levels at four power generation states and three measurement stations: Station 1 (110 m range; top row), Station 2 (170 m range; middle row), and Station 3 (10 m range; bottom row).

**Fig. 3:**
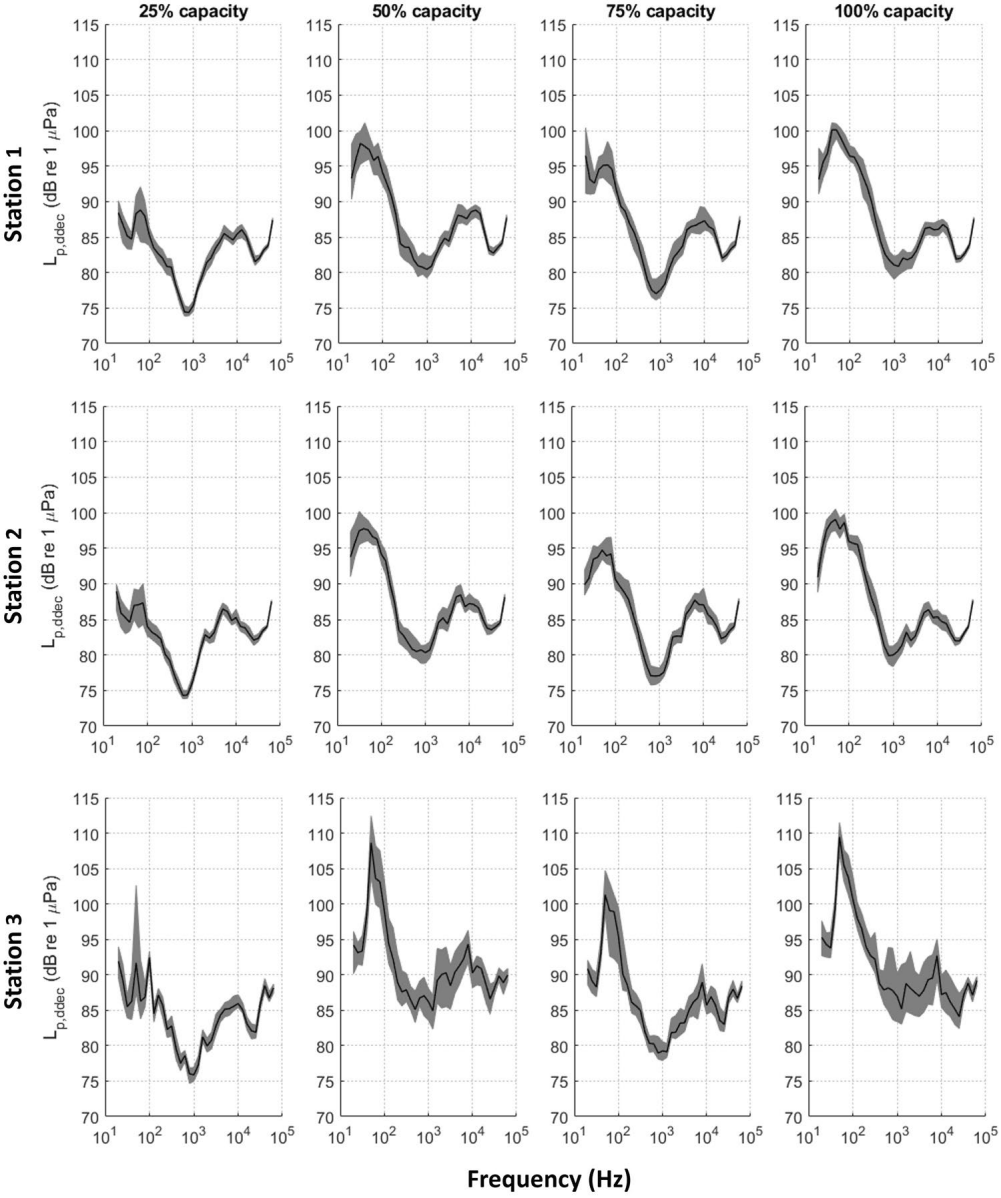
Median values (black curves) and interquartile range (gray shadow) of the decidecadal sound pressure levels at four power generation states and three measurement stations: Station 1 (170 m range; top row), Station 2 (110 m range; middle row), and Station 3 (10 m range; bottom row).

The −40 TS guidance for a Level A characterization aims to quantify WEC sound emissions across a range of temporal and spatial scales. Figures 1-3 show the data products described by the −40 TS. Figure 1 shows the median WEC associated sound pressure levels (*SPL*; 10 Hz - 96 kHz) as a function of significant wave height ($H_s$) and wave energy period ($T_e$) measured at each of the three hydrophone stations around the x1™ WEC. At all three stations, median *SPL* increased with rising $H_s$. This can likely be attributed to higher breaking wave acoustic intensity^34^. Station 3, the closest hydrophone to the WEC at a distance of 10 m, recorded higher median *SPL*s than Stations 1 and 2 (110 and 170 m ranges, respectively) at all measured wave conditions. At Station 3, median *SPL* increases with $H_s$, yet decreases with $T_e$ across the entire range of measured sea states. This suggests higher intensity acoustic emissions from a device may not be directly related to the energy density of the incident wave field, but rather how the WEC controls are optimized and tuned to operate in specific conditions that may generate more mechanical noise. Furthermore, the patterns observed at Station 1 and 2 are nearly identical, suggesting much of the low frequency acoustic energy from the WEC observed at Station 3 had attenuated at ranges of 110 m and 170 m.

Figure 2 quantifies the spectral characteristics of WEC sound emissions through discrete levels of the normalized power capacity during the 4 month recording period. Mean-square sound pressure spectral density levels $L_{p,f}$ at four discrete power generation states (25%, 50%, 75%, 100% of the WEC rated capacity) are shown for the 3 hydrophone stations. The median $L_{p,f}$ values are plotted with their corresponding interquartile ranges (25*th*−75*th* percentiles) to indicate the amount of variability observed within a particular frequency. The spikes observed in the spectral levels above 10 kHz are electrical line noise that is inherent to the hydrophone systems. Stations 1 and 2 maintain a relatively consistent spectral shape across the power generation states with slightly elevated low frequency energy at higher power generation. At 25% capacity, $L_{p,f}$ median energy levels at frequencies < 100 Hz remain between 70–80 dB re 1 $\mu \textrm{Pa}^2\,\textrm{s}$, while at the higher power generation states, they are between 80–90 dB re 1 $\mu \textrm{Pa}^2\,\textrm{s}$. A notable drop in received levels is observed near 1 kHz at 25% that is replaced by increased energy levels at the higher capacity ratings. At the highest power generation state of 100%, a change in the spectral slope is observed as spectral levels increase ranging from a few hundred Hz to tens of kHz. The Station 3 median values and interquartile ranges reveal a low frequency spectral peak below 100 Hz with energy levels between 90–100 dB re 1 $\mu \textrm{Pa}^2\,\textrm{s}$. This peak maintains a consistent spectral structure across all power generation states, and has the highest levels of acoustic intensity at the maximum power generation state. The Station 3 recordings exhibit higher intensity “spikes” or discrete spectral peaks in the $L_{p,f}$ values at higher frequencies (> 10 kHz) at all power generation states. These higher intensity spikes are associated with electrical line noise of that particular hydrophone system.

Similar to the spectral characteristics shown in Figure 2 at 1 Hz resolution, median and interquartile values averaged over decidecadal frequency bands are shown for each rated power generation state and each hydrophone station in Figure 3. The decidecade mean-square sound pressure spectral density levels ($L_{p,ddec}$) highlight the similarities in the acoustic energy received at Stations 1 and 2 across the power generation states. Meanwhile, Station 3 $L_{p,ddec}$ levels reveal the low frequency WEC generated acoustic peak with energy up to 10 dB re 1 $\mu \textrm{Pa}^2\,\textrm{s}$ higher than was recorded in that frequency band for Stations 1 and 2. Interestingly, at Station 3, the intensity of this peak was lower for the 75% power generation state than 50% or 100%, respectively.

**Fig. 4:**
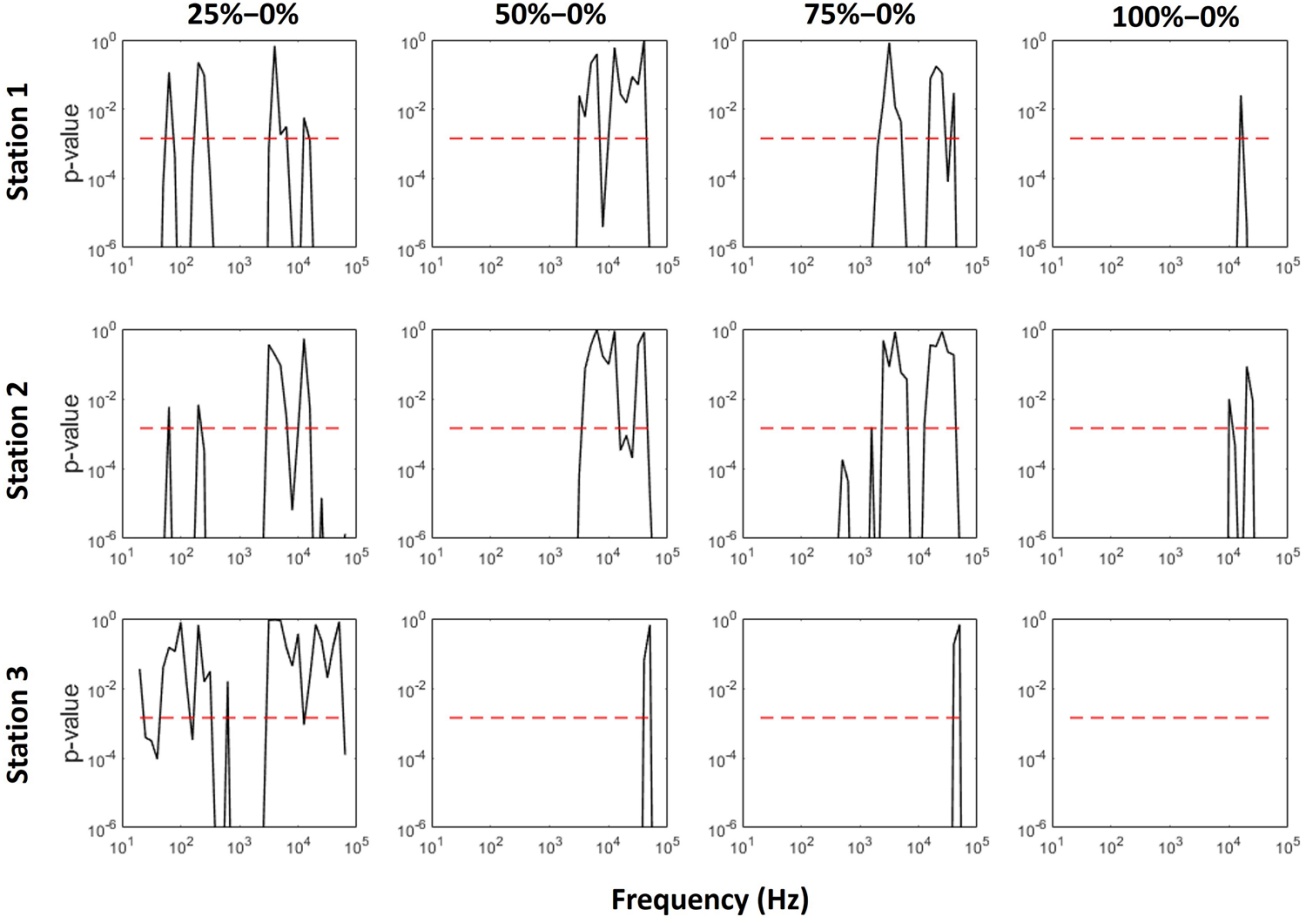
P-values of pair-wise Mann-Whitney tests for all decidecadal bands comparing wave energy converter at 25%, 50%, 75% and 100% of the rated capacity to ambient noise (0% capacity) at Station 1 (top row), Station 2 (middle row), and Station 3 (bottom row). Red-dash lines indicate 5% significance level.

Although not a component of the −40 TS guidance, pair-wise Mann-Whitney tests were applied for statistical comparisons between decidecadal sound pressure spectral density levels ($L_{p,ddec}$) measured at each WEC power generation state and the ambient conditions (0% WEC power) for each hydrophone^28^. At Station 1 (Figure 4a-d) and Station 2 (Figure 4e-f), p-values remain below the 5% significance level in frequencies below 1 kHz for 50%, 75%, 100% capacities indicating sound in these frequencies and power generations states are statistically different than ambient conditions. At Station 3 (Figure 4i-l), for capacity ratings 50%, 75%, 100% p-values show $L_{p,ddec}$ levels are statistically different from ambient levels at nearly all measured frequencies, while at 25% capacity only a few frequencies below 100 Hz and around 1 kHz show significant differences from ambient conditions.

### Regulatory and conservation management criteria informed by acoustic characterization

The U.S. National Marine Fisheries Service (NMFS) developed a cumulative sound exposure level ($SEL_{cum}$) framework with weightings specific to five animal hearing groups that is used by regulators in the U.S. to assess the potential onset of auditory injury or temporary threshold shifts for marine mammals from acoustic emissions^19^. The five hearing groups are:low frequency LF cetaceans (baleen whales) 7 Hz - 36 kHz; high frequency HF cetaceans (dolphins, toothed whales, beaked whales, bottlenose whales) 15 Hz - 160 kHz; very high frequency VHF cetaceans (true porpoises, river dolphins, *Kogia*, cephalorhynchid) 200 Hz - 165 kHz); Phocid pinnipeds PW (true seals) 40 Hz - 80 kHz; and Otariid pinnipeds OW (sea lions and fur seals) 60 Hz - 68 kHz. The upper limits of the hearing ranges of the HF and VHF and the lower limits of the LF and HF groups extend beyond the frequencies measured in this study (20 Hz-96 kHz), so our $SEL_{cum}$ measurements are limited to the measured range. However, based on previous WEC measurements, as well as those shown here, it is unlikely that WEC generated sounds extend beyond the −40 TS default frequency limits of 10 Hz and 100 kHz. Therefore, measurements result in a robust acoustic characterization of WEC emissions for application to the U.S. NMFS threshold criteria across all of the hearing group frequency ranges.

The U.S. NMFS framework is based on weighted cumulative sound exposure levels over a 24 hour period ($SEL_{24h}$) to evaluate both auditory injury and temporary threshold shifts (TTS) from non-impulsive sounds in each of the animal hearing groups. Taking a conservative approach for potential acoustic effects, we applied the hearing group weightings to the WEC power generation state with the highest *SPL* in each hearing group to integrate over a 24 hour period at each hydrophone station. In Table 1, the weighted cumulative sound exposure $SEL_{24h}$ values for a 24 hour period for each hydrophone and hearing group are compared with the thresholds for auditory injury and TTS defind by the U.S. NMFS. Even using the WEC power state with the highest *SPL* from each station, the calculated weighted $SEL_{24h}$s do not approach levels for TTS or Auditory Injury with the closest being the low frequency hearing group for Station 3 at nearly 20 dB below the threshold for TTS. If $SEL_{24h}$ were calculated with *SPL*s measured during lower WEC power generation states, this difference would only increase.

**Table 1:** Weighted cumulative sound exposure levels (dB re 1 $\mu Pa^2 s$) over 24 hours for 5 animal hearing groups at three hydrophone stations, following the U.S. NMFS auditory weighting functions and the thresholds for temporary threshold shift (TTS) and auditory injury (Aud Inj)^19^.

Hearing Group	Station 1	Station 2	Station 3	TTS	Aud Inj
LF (7 Hz - 36 kHz)	154.8	153.7	158.9	177	197
HF (15 Hz - 160 kHz)	149.7	148.4	153.9	181	201
VHF (200 Hz - 165 kHz)	148.0	146.4	151.7	161	181
PW (40 Hz - 80 kHz)	150.0	148.9	154.1	175	195
OW (60 Hz - 68 kHz)	148.1	146.8	152.6	179	199

**Fig. 5:**
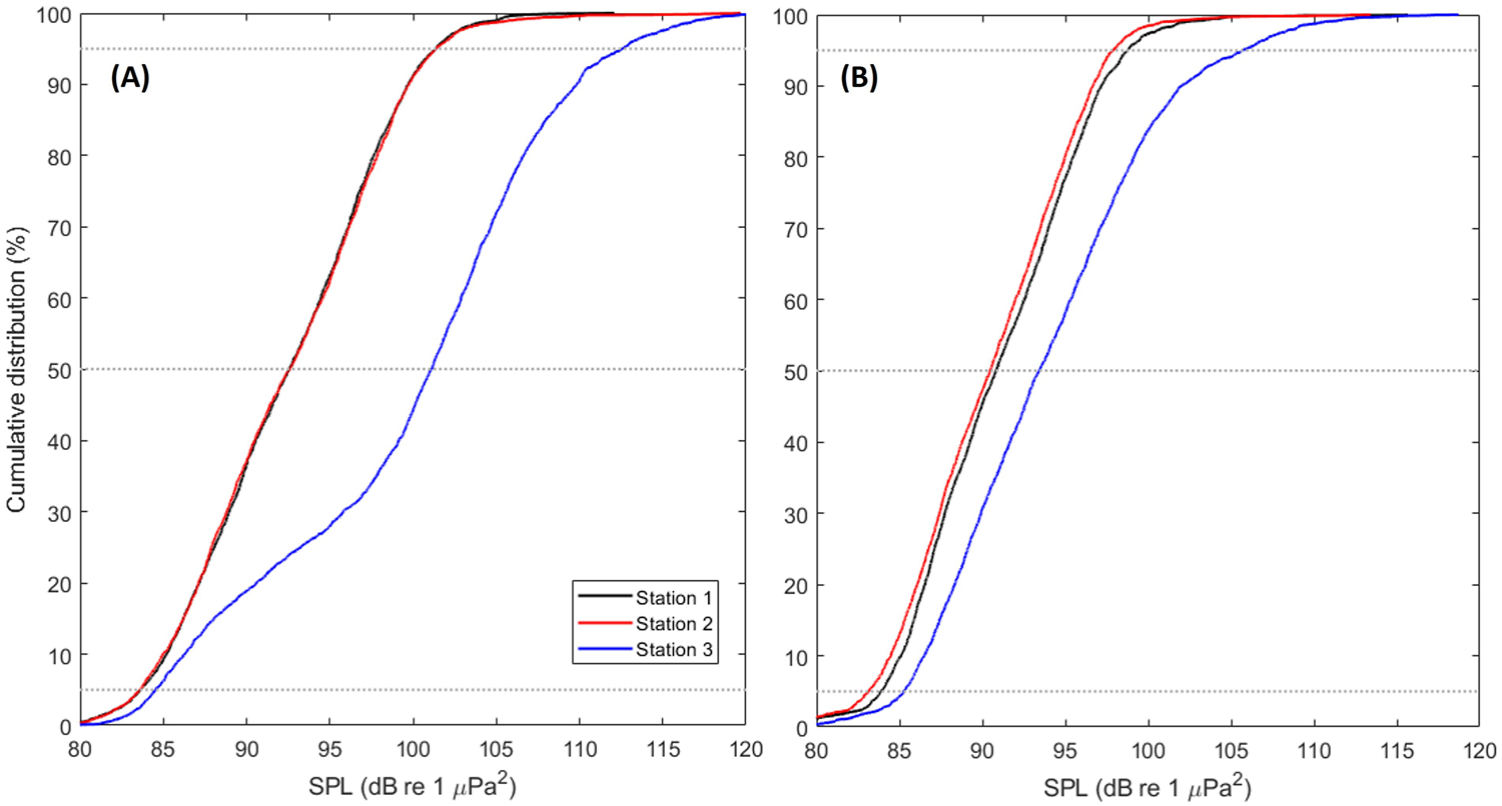
The cumulative distribution of WEC associated median sound pressure levels (*SPL* over 30 second windows across all of the WEC power generation states) calculated for 1/3 octave bands centered at 63 Hz (Panel A) and 125 Hz (Panel B) at Station 1 (black), Station 2 (red), and Station 3 (blue). Dotted lines denote the 5%, 50%, and 95% percentiles.

Conversely, the E.U. Marine Strategy Framework Directive (MSFD) addresses continuous low-frequency noise in the 63 Hz and 125 Hz one-third-octave bands, and suggests the use of cumulative distribution functions to present the distribution of sound pressure levels in these bands. These are shown for samples when the WEC was operating in Figure 5. Here, the cumulative distributions of sound pressure levels in these bands highlight the differences between Station 3 and Stations 1 and 2. The most pronounced differences were observed in the third octave band centered at 63 Hz (Figure 5A) - sound pressure levels at the closest hydrophone (Station 3, 10 m) were at least 8 dB higher than those at the more distant stations (Station 1 and 2, 110 and 170 m) more than 70% of the time. Below the 25th percentile (representing 25% of the time), the difference between the stations decreases, reflected as a change in the cumulative distribution slopes. Further, the cumulative distribution curves for Stations 1 and 2 are nearly equivalent for the 63 Hz third octave band, suggesting similar received levels despite a 60 m difference in distance from the WEC. In the third octave band centered at 125 Hz (Figure 5B), the difference between the cumulative distribution functions for Station 3 and the more distant stations remains evident, and increases at the highest percentiles. The offset between the near and far stations drops to a difference of less than 5 dB at the median (50th percentile). Unlike the 63 Hz band, 125 Hz band levels at Station 1 are consistently slightly higher (< 1 dB) than at Station 2, (Table 2).

**Table 2:** Sound pressure levels (dB re *1 μ Pa*) from third octave bands centered at 63 Hz and 125 Hz at the 5%, 50%, and 95% percentile cumulative distribution values.

	63 Hz			125 Hz		
Station	5%	50%	95%	5%	50%	95%
1	83.6	92.6	101.4	83.9	90.9	98.8
2	83.6	92.6	101.4	83.1	90.5	97.9
3	84.6	101.1	112.6	85.3	93.4	105.7

## Discussion

**Fig. 6:**
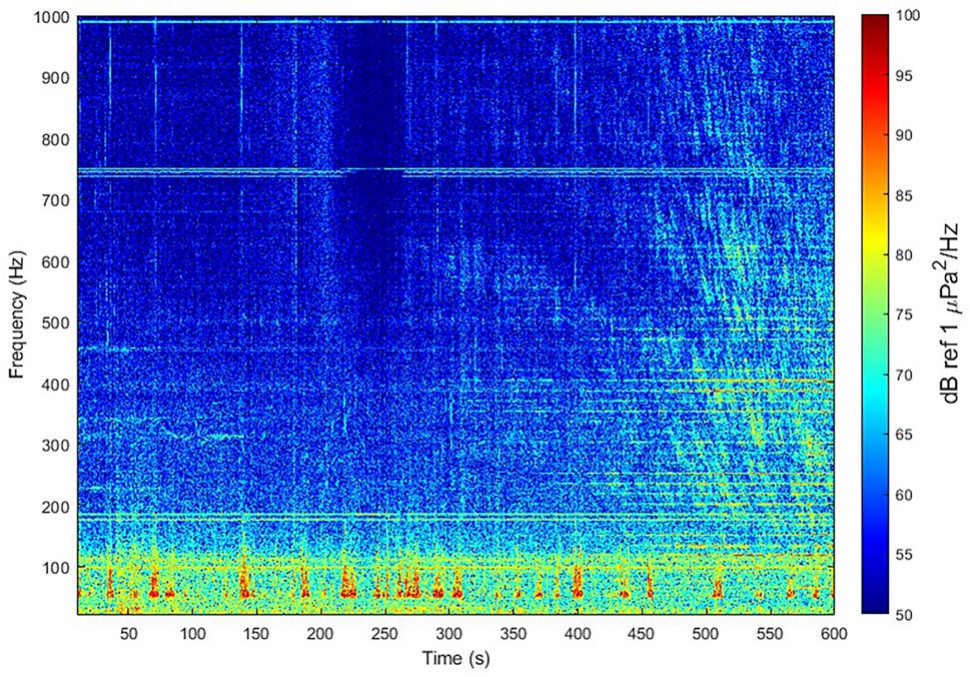
An example spectrogram showing failed acceptance criteria from vessel traffic acoustic emissions that contaminate the sample recording for WEC isolated sounds.

The −40 TS guidance provides a method for standardized characterization of marine energy converter sound emissions. An important component of the data processing includes global sample acceptance criteria that ensures data sequences with sound sources other than the WEC are excluded from the analyses. From the criteria described in the −40 TS, samples were manually reviewed by visual inspection of the spectrogram, hydrophone voltage time series, and auditory playback. As per the −40 TS guidance, samples were excluded if they contained obvious non-WEC anthropogenic sounds (e.g. vessel traffic), non-WEC biological sounds (e.g. whale calls), hydrophone voltage saturation, or mooring platform related noise. An example of a sample that failed the acceptance criteria is shown in (Figure 6) where acoustic emissions from an approaching vessel are readily observed in the later half of the spectrogram (> 300 seconds).

Because of the global acceptance criteria, the −40 TS characterization is limited to only WEC sounds and does not include contamination from other transient sources that might occur in the soundscape. This is crucial for accurate representation of WEC sound in regulatory frameworks because it enables singular evaluation of the acoustic emissions of the WEC, independent of the surrounding soundscape. The approach promotes transferability of the WEC acoustic characterization to other similar environments, and, at a minimum provides stakeholders with quantitative expectations for the WEC’s acoustic emissions through a range of operation and sea states. To our knowledge, this work presents the first attempt at translating results derived from the −40 TS methodology to the metrics and data products used by regulators assessing the potential environmental impacts of marine energy devices.

The application of the −40 TS methodology and translation to the U.S. regulatory criteria for the observed sea and power generation states of the CalWave x1™ WEC test near San Diego, California in 2022 show that sound emissions were well below U.S. NMFS threshold criteria for temporary threshold shifts and auditory injury to all marine mammal hearing groups at all measured distances ranging from 10 m - 170 m from the device. Because the data products derived from the −40 TS analysis comprehensively described the sound generated by the WEC, calculation of the metrics required for this analysis ($SEL_{24h}$) were straightforward.

From the EU monitoring framework perspective, the −40 TS results facilitated evaluation of the acoustic contribution of the WEC and capture its spatial decay pattern across relevant MSFD third-octave bands centered at 63 Hz and 125 Hz. Notably, the cumulative distribution curves for 63 Hz indicate that beyond a certain distance from the device, received levels tend to stabilize, likely reflecting the combined effect of transmission loss and environmental factors that reduce measurable differences between stations. For the 125 Hz band, the differences between the three Stations remain similar, though less pronounced. Overall, the median sound pressure level cumulative distributions for both frequency bands suggest a relatively confined low frequency acoustic footprint of sound emissions around the WEC.

Because the −40 TS methodology characterizes the WEC as an isolated acoustic source, application of the results do not account for contributions from geophony, biophony, and other anthropogenic sources required for the holistic soundscape impact assessment approach taken by the EU. However, the information that the −40 TS provides can be useful from a EU legislative perspective. From a regulatory point of view, the Marine Strategy Framework Directive (MSFD) adopts a dynamic, ecosystem-based approach that does not prescribe fixed acoustic threshold criteria. Instead, the EU Technical Group for Underwater Noise guidance^33^ requires that no more than 20% of species habitat should be exposed, within any month of an assessment year, to noise level exceeding the Level of Onset Biologically Adverse Effects (LOBE), in line with conservation objectives related to maintaining 80% of the habitat carrying capacity. This approach explicitly recognizes that species and habitats differ in their sensitivity to sound exposure, and that the overall background noise varies in space and time. Consequently, assessment is not based on passing or failing a single numerical threshold, but on determining whether biologically relevant criteria are exceeded across meaningful spatial and temporal scales. More broadly, the MSFD framework promotes the adoption of quieting technologies and management measures such as time–area restrictions.

Earlier European studies dealing with ocean renewable energy systems focused on acoustic threshold exceedances and masking effects associated with their emissions, without directly referring to MSFD guidelines^28,31^. More recent studies from deployed WECs or Offshore Wind Farms (OWFs) in European waters have incorporated the MSFD frequency bands into their analysis and report temporal variability over the monitoring period^35,36^. Notably, Baldachini et al., (2025)^37^, in evaluating the introduction of renewable energy systems into EU marine environments, refers to internationally recognized biological thresholds also adopted by the U.S., even though these are not formally embedded in European regulations.

The results of the −40 TS analysis provide valuable information for assessment under the MSFD, and, like the NMFS guidelines, make calculation of the recommended metrics straightforward. However, specific guidelines to directly apply MSFD requirements to ocean renewable energy are lacking. The spatio-temporal evaluation framework adopted in the EU takes a holistic ecosystem approach that is not based on threshold criteria, and requires additional information beyond the −40 TS single source or array based characterization for determining potential acoustic impacts.

It is important to note the results from the single prototype scale device used in this case study example are not reflective of commercial or array scale deployments of marine energy devices in other environments. Rather, this study provides an example that exercises the −40 TS for an operational WEC in an effort to help encourage standardized acoustic measurements at future deployments. Additionally, we demonstrate how the acoustic characterization outputs from the −40 TS can be translated to help inform regulatory criteria in the US and EU.

## Methods

### CalWave $\hbox {x1}^{\textrm{TM}}$ WEC and test site description

**Fig. 7:**
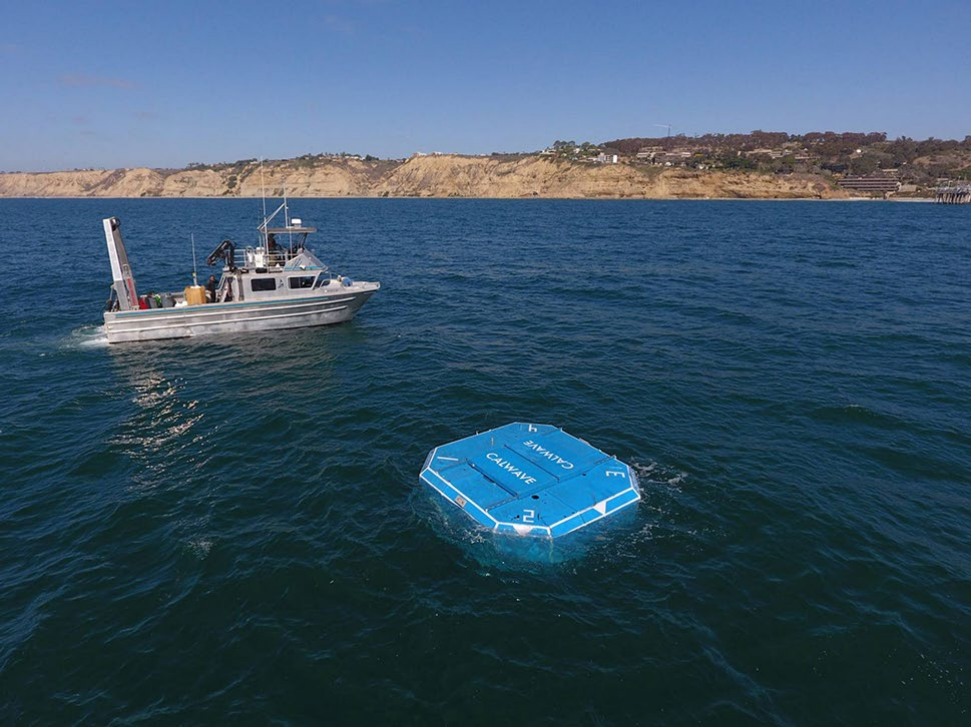
CalWave’s x1™ prototype wave energy converter deployed in 20 m water depth near the Scripps Institution of Oceanography pier in La Jolla, California U.S. (photo credit: CalWave).

**Fig. 8:**
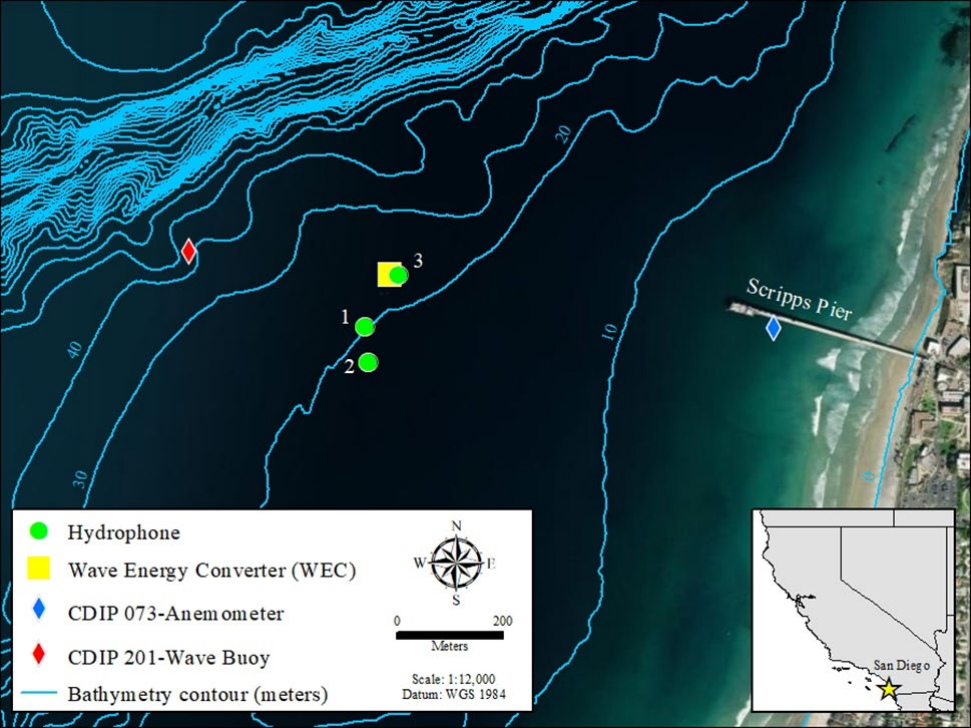
A map of the CalWave x1™ wave energy converter project site and acoustic characterization study near the Scripps Institution of Oceanography pier. Depth contours are shown in 10 m intervals. Inset map in the lower right shows the general location of the project in southern California.

In September 2021, the wave energy developer CalWave deployed a point absorber device, the x1™ (Figure 7), in 25 m of water at a location 1500 m west of the Ellen Browning Scripps Memorial Pier in La Jolla, California (Figure 8). The x1™ is a scaled prototype version of CalWave’s x1000™ 1000-kilowatt device and relies on the pressure differential created by passing waves to create motion. Generators inside the WEC convert the resulting reaction forces between the device body and its mooring into electricity. Power from the x1™ was delivered to shore by an umbilical cable deployed on the seafloor that connected the device to Scripps Pier and a shore station. The horizontal position of the WEC was maintained using a taut-line mooring system connected to four, equally dispersed gravity anchors. The x1™ operated continuously from September 2021 with over 99% system uptime from November 2021 until it was recovered in July 2022.

The site has a sandy bottom and is exposed to open ocean swells. The wave climate and wind speeds were measured by the nearby Scripps Nearshore wave buoy (CDIP 201; Figure 8) and the Scripps Pier meteorological station (CDIP 073; Figure 8).

### Acoustic data collection

**Fig. 9:**
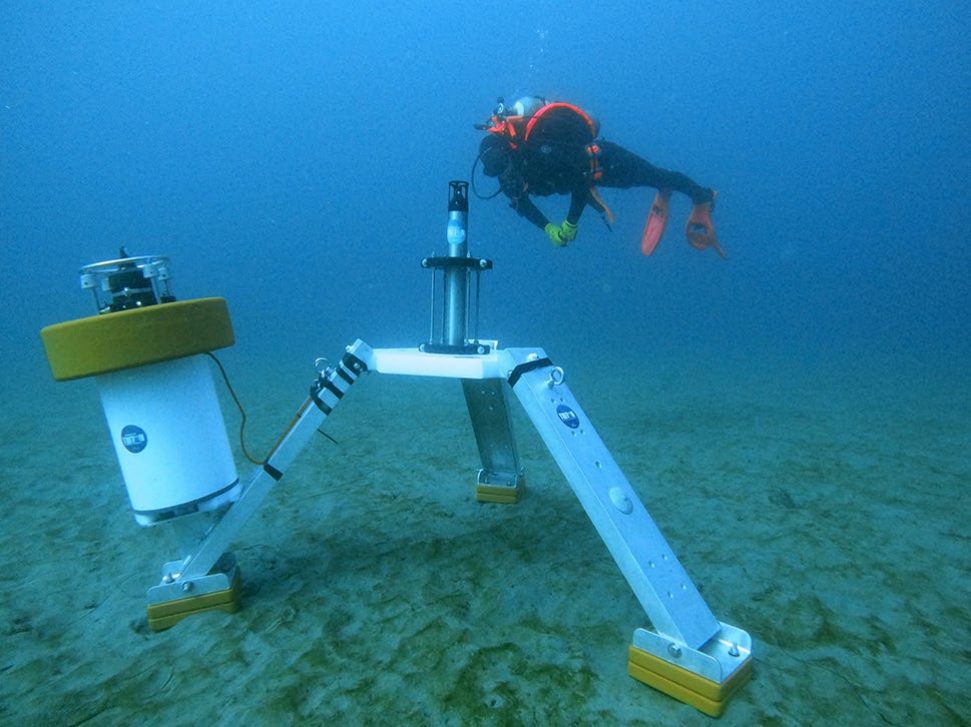
An underwater image of the DeepWater®Buoyancy bottom lander equipped with a SoundTrap™ hydrophone (center) and pop up buoy recovery system (left) deployed on the seafloor near the x1™ wave energy converter (photo credit: Richard Marsh).

Three Ocean Instrument Soundtrap™ ST600 HF long term recording hydrophones were mounted on DeepWater Buoyancy BTM-AL50-B™ tripod bottom landers with the sensors 1 m above the lander base (Figure 9) and were deployed near the x1™ from R/V Bob and Betty Beyster on February 1, 2022. The hydrophone landers had a submerged weight of 60 kg and were equipped with DeepWater Buoyancy Pop Up Buoy™ recovery systems each outfitted with an EdgeTech PORT LF-SD™ acoustic release transponder. The hydrophones were configured to record continuously for 10 minutes every 20 minutes on a 50% duty cycle at a sample rate of 192 kHz. All three SoundTrap™ hydrophones successfully recorded data from February 3, 2022 through their recoveries on May 19, 2022 for a total of 106 recording days. Prior to deployment, hydrophones were calibrated for low frequencies 1–700 Hz at Ocean Networks Canada and high frequencies 50,000–100,000 Hz at the Pacific Northwest National Laboratory accredited facility.

Following the −40 TS guidance for positioning, two bottom landers were deployed in alignment with the dominant winter northerly swell direction of the site (Figure 8). Station 1 and Station 2 were separated from the WEC by a horizontal distance of 110 m and 170 m and were located at depths of 20 m and 19 m below MLLW, respectively. The deployment positions of Station 1 and Station 2 deviated from the −40 TS suggested 100 m and 200 m offsets from the WEC in order to avoid other research equipment in the area. A third hydrophone lander (Station 3) was deployed inshore 10 m east from the WEC at a depth of 24 m below Mean Lower Low Water and perpendicular to the dominant northerly swell direction. After initial deployment from the support vessel, the Station 3 final position was adjusted by divers to a distance 10 m and a direct line of sight to the x1™ preventing acoustic shadowing from the large nearby WEC gravity anchors. Moving Station 3 in closer to the WEC and deviating from the −40 TS 100 m standoff distance was done in an effort to better capture and characterize signals from the x1™ after previous measurements revealed low amplitude acoustic emissions (Raghukmar pers comm).

Before the hydrophone systems were recovered on May 19, 2022, an acoustic survey of the landers was carried out to provide more precise locations of each station. A surface GNSS receiver was placed above an underwater transducer suspended below the vessel hull. An EdgeTech PACS™ acoustic deck box was used to interrogate the slant range to the underwater acoustic release transponder mounted on each of the hydrophone landers. A field computer simultaneously recorded the time synced GNSS location and acoustic range data while the vessel slowly circled each lander providing up to 50 range measurements. An open-source python package called OBSRange^38^ was then used to calculate the 3D location of each lander, with horizontal errors estimated between 1–2 m. Water temperature, salinity and soundspeed depth profiles were collected using a Sontek CastAway™ during the deployment and recovery operations of the hydrophone landers on February 3 and May 17, 2022. No significant differences in sound speeds were observed.

### Acoustic data processing

A valuable component of the −40 TS processing and analysis is the reduction of the larger acoustic data record to a smaller subset that is singularly focused on WEC sound emissions. Global sample acceptance criteria require manual visual review of spectrograms to exclude periods with non-WEC anthropogenic sounds (e.g. vessels), biological sounds (e.g. whale calls), or system self-noise (e.g. mooring sounds). For the Level A characterization, acoustic measurement sequences consist of a 30 second window of continuous samples with at least 80% of 1 second intervals within the 30 second window meeting acceptance criteria. A minimum of 20 valid acoustic measurement sequences is required for each sea state bin (Figure 1). These acoustic measurement sequences are then linked to the power generation time series of the WEC to connect the wave conditions and power generation state of the device with its acoustic emissions.

**Fig. 10:**
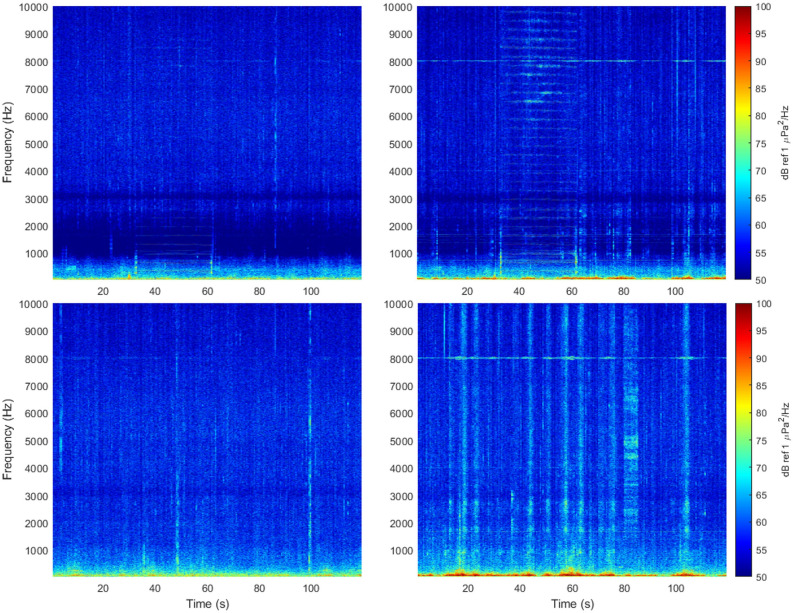
Spectrograms (20 Hz - 10 kHz) showing the two dominant WEC sounds identified in the hydrophone records on Station 1 (left) and Station 3 (right). WEC sound 1 is shown in the top panels and WEC sound 2 is shown in the bottom panels.

**Fig. 11:**
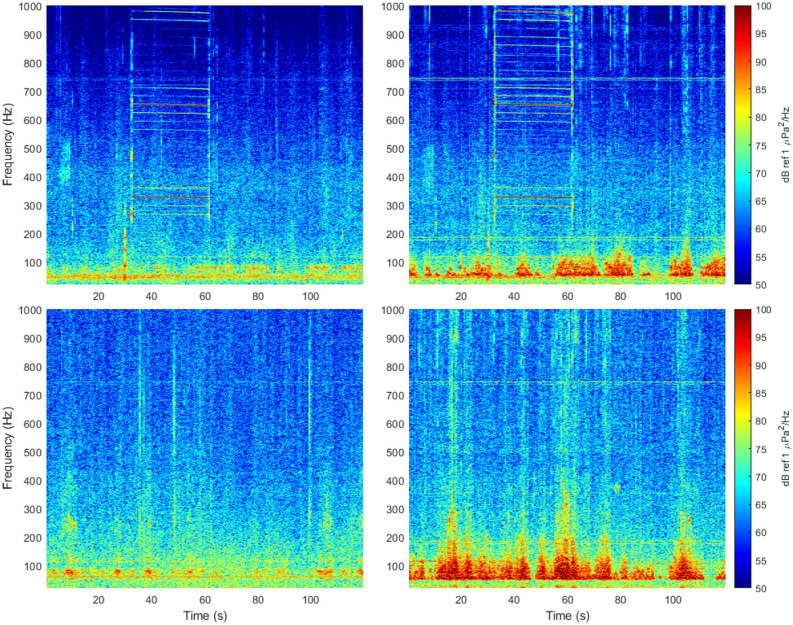
Low frequency spectrograms (20 Hz - 1 kHz) showing the same two dominant WEC sounds from Figure 10 with Station 1 (left) and Station 3 (right). WEC sound 1 is shown in the top panels and WEC sound 2 is shown in the bottom panels.

Attributing acoustic signals to a WEC can be challenging in the midst of a complex coastal soundscape. Fortunately, the global acceptance criteria of the −40 TS helps by providing a general methodology based on manual review for rejecting samples. From accepted sample recordings around the CalWave x1™ WEC, we identified two types of repetitive acoustic emissions. Figure 10 shows spectrograms of the two types of WEC sounds observed at Station 1 (left) and Station 3 (right). WEC sound 1 (upper panels) consists of a series of 30 second long continuous tones with harmonic spectral structure that extends above 10 kHz. This sound is characteristic of acoustic modem transmissions and may be related to an operational control or communication component of the x1™ test. WEC sound 2 (lower panels) consists of wideband energy that extends from a peak at 50–54 Hz to above 10 kHz with much higher WEC operational related acoustic energy than WEC sound 1. Focusing on the lower frequency components of this sound Figure 11 shows that these signals exhibit significantly higher received levels at Station 3 located 10 m from the WEC than at Station 1, 110 m away. There is also a persistent tone at *≈*8 kHz associated with the WEC and observed on both Stations but with stronger intensity at Station 3 than at Station 1.

## Data Availability

Data will be made available under license CC-Attribution 4.0 via the Portal and Repository for Information on Marine Renewable Energy (PRIMRE) on the Marine and Hydrokinetic Data Repository (MHKDR) [https://mhkdr.openei.org/].
